# Discrete and dimensional approaches to affective forecasting errors

**DOI:** 10.3389/fpsyg.2024.1412398

**Published:** 2024-07-23

**Authors:** Prsni Patel, Heather L. Urry

**Affiliations:** Department of Psychology, Tufts University, Medford, MA, United States

**Keywords:** affective forecasting, affective forecasting errors, measurement, discrete emotions, dimensional affective states

## Abstract

Evidence for affective forecasting errors is mixed. We review recent studies to determine whether taking a discrete versus dimensional approach to measuring affective forecasting could partly explain this inconsistency. We observed variation in measurement approaches to measuring and analyzing affective forecasting; those that adopted a discrete approach often examined high arousal positive (e.g., excitement) and negative (e.g., anger) emotions. We recommend conducting empirical studies and meta-analyses to examine whether affective forecasting errors differ systematically depending on measurement approach. Furthermore, we recommend expanding the scope of affective forecasting investigations to examine more granular dimensional affective states and low-arousal discrete emotions. The ideas and future directions presented enhance our understanding of affective forecasting errors and how we study them.

## 1 Introduction

The process of making predictions about how one will feel in the future is known as affective forecasting (Wilson and Gilbert, 2003). Most affective forecasting research has focused on the idea that people tend to inaccurately overestimate the intensity and duration of their future emotions (Gilbert et al., 1998; Wilson et al., 2000). For instance, people overestimate how nervous they will feel when running a race (Aitken et al., 2021) and how much negative affect they will feel when their preferred candidate loses the presidential election (Barber et al., 2023). This tendency to inaccurately overestimate the intensity and duration of one’s future emotions, or the impact bias, has been the focus of affective forecasting research for over a decade (e.g., Gilbert et al., 1998; Dunn et al., 2007; Hoerger et al., 2010). Sixty-six percent of the articles in two meta-analyses on affective forecasting (Levine et al., 2012; Mathieu and Gosling, 2012) focused on forecasting inaccuracy, using keywords in their titles such as error, bias, and failure (Hoerger et al., 2016).

The conclusion that people generally make affective forecasting errors about the intensity of their future emotions, however, is too simple.^1^ There is variation across studies of affective forecasting errors. In particular, while some studies have found that people overestimate the intensity of their future affect, others have found that people sometimes *underestimate* future intensity (e.g., Lench et al., 2011; Ruby et al., 2011; Zelenski et al., 2013), or that people can also make *accurate* affective forecasts (e.g., Levine et al., 2012; Lench et al., 2019). Findings are, thus, inconsistent.

Understanding the reasons for inconsistent findings is crucial for theoretical and practical reasons alike. Theoretically, understanding the sources of inconsistencies can inform the inferences that researchers make from their studies and highlight gaps in the research that future studies can fill. In particular, investigating potential sources of inconsistent findings represents a crucial first step, in that it lays the foundation for future studies to empirically examine the conditions under which affective forecasting errors emerge. Practically, affective forecasts are pervasive in people’s everyday lives; they may guide the situations that people choose to immerse themselves in (e.g., Urry and Gross, 2010), influence performance on tasks (e.g., Kaplan et al., 2020), and decision-making in domains such as healthcare (e.g., Hoerger et al., 2016) and travel (e.g., Karl et al., 2021). Thus, better understanding the source of affective forecasting errors can improve our understanding of their effects on these downstream processes.

Past researchers have examined two potential sources of variation in findings about affective forecasting errors for the intensity of future emotion (Levine et al., 2012; Mathieu and Gosling, 2012). Specifically, Levine et al. (2012) found that when people were asked to imagine an event, make forecasts about how they would feel, and later report how they actually felt *in reference to that event*, they made relatively accurately predictions as opposed to when they were asked to imagine an event, forecast how they would feel, and later report how they felt in general, *without any reference to the event*. Additionally, Mathieu and Gosling (2012) found through a meta-analysis that when researchers adopted an “absolute” approach (i.e., computed the difference between forecasted and actual affect), people were inaccurate at predicting their emotions, as opposed to when they adopted a “relative” approach (i.e., computed the correlation between forecasted and actual affect).

In this mini review, we focus on a novel source of variation – the divergent measurement approaches used in affective forecasting studies. Accordingly, we first describe two broad theoretical approaches to emotion research, discrete and dimensional. Arguably, researchers’ emotion theories guide their corresponding measurement approaches. We then selectively review studies from the last few years to understand the extent to which researchers take discrete and dimensional approaches to measuring affective forecasting errors for emotion intensity and the existing gaps in assessment. We conclude with recommendations for future research that will move the field forward in understanding the extent to which affective forecasting errors vary systematically as a function of discrete and dimensional measurement approaches.

## 2 Discrete and dimensional approaches to affective forecasting

Broadly speaking, emotion researchers typically adopt a discrete or dimensional approach to emotion – based on the theory of emotion with which they are most closely aligned. According to the basic/discrete emotions theory, humans have evolved to have a set of basic emotions in response to threats and challenges in their environments (Ekman, 1992; Tooby and Cosmides, 2008). This model proposes three main features of emotions – first, that they have evolved to serve distinct adaptive functions. For example, the emotion of fear is believed to have evolved to help us flee predators and other sources of threat (Öhman and Mineka, 2003). Second, each discrete emotion has its own unique neural pathway in the central nervous system that, once activated, leads to its own signature profile of physiology, behavior, and cognition (Posner et al., 2005). Continuing with the example of fear – it activates one specific neural pathway that leads to a racing heartbeat, increases in skin conductance, widening of the eyes, and other overt behaviors. Lastly, discrete/basic emotion theorists believe that while people across cultures might interpret emotions slightly differently and even create their own emotion concepts, certain core emotions are innate and thus universal across people and cultures (Ekman and Friesen, 1971). For instance, the emotion of fear was identified by people from New Guinea who had had little to no exposure to Westerners or Western culture (Ekman and Friesen, 1971).

Accordingly, researchers who adopt the discrete emotions approach measure and analyze each emotion as its own category. For instance, in the affective forecasting literature, Aitken et al. (2021) asked participants to rate how much excitement, confidence, pride, frustration, and nervousness they expected to experience and actually experienced. Subsequently, they conducted separate paired samples t-tests for *each* discrete emotion to examine mean differences in predicted and actual intensity.

**Figure 1:**
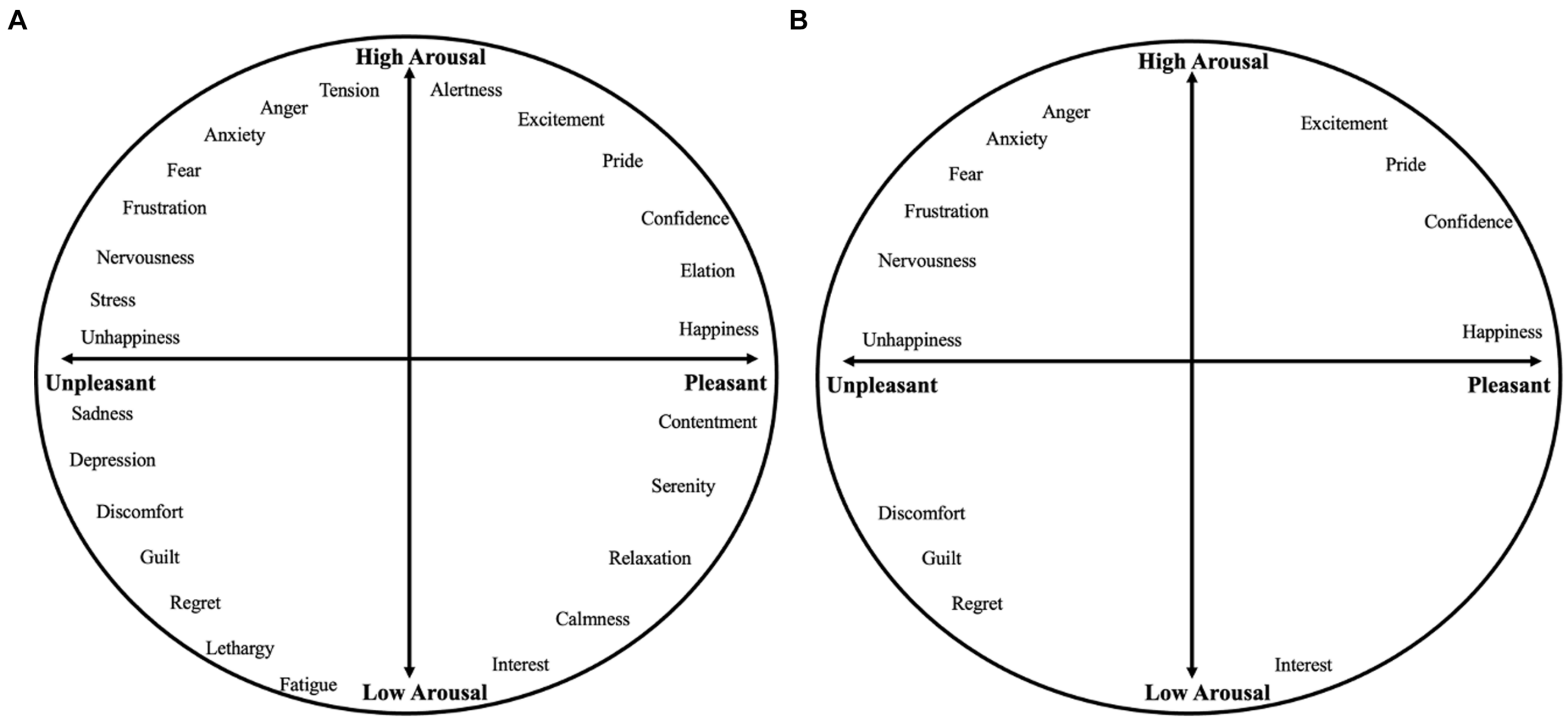
**(A)** Affective circumplex (adapted from Russell, 1980) displaying valence (*x*-axis) and arousal (*y*-axis). **(B)** Affective circumplex illustrating the discrete emotions that affective forecasting researchers have examined within the last few years.

By contrast, researchers who adopt the dimensional (or core affect) theory of emotion, conceptualize emotions as combinations of broader underlying processes or dimensions. While there are several two-dimensional models [e.g., positive and negative affect (Watson et al., 1999), approach and withdrawal (Lang et al., 1998)], here we consider the affective circumplex model, comprising dimensions of valence and arousal (Russell, 1980) (see Figure 1). In this model, valence, as displayed on the x-axis, refers to the level of unpleasantness to pleasantness, and arousal, as seen on the y-axis, refers to the level of activation one experiences. Accordingly, each emotion is a linear combination of some level of valence and arousal. For instance, fear is an emotion that is conceptualized as a combination of negative valence and high arousal (Posner et al., 2005). Hence, fear is situated in the upper left quadrant of Figure 1, along with other high arousal negative emotions such as anger and frustration. The upper right quadrant comprises emotions that are a combination of high arousal and positive valence such as excitement and elation. The lower half of this circumplex contains the low arousal negative quadrant including emotions such as guilt, and regret, and the low arousal positive quadrant including emotions such as contentment and calmness.

Researchers who adopt the dimensional approach may measure and analyze valence and/or arousal directly. For instance, in the affective forecasting literature, Aitken et al. (2021) measured participants’ predicted and actual valence and arousal. They then analyzed mean differences in predicted and actual valence and arousal using paired samples t-tests. Alternatively, researchers aligned with the dimensional approach may measure several discrete emotions and combine them into composite indices of positive and negative affect. For instance, Barber et al. (2023) measured predicted and actual levels of three discrete low arousal positive (calm, relaxed, content) and negative emotions (bored, lonely, sluggish), and three discrete high arousal positive (excited, enthusiastic, activated) and negative emotions (angry, anxious/worried, disappointed). Subsequently, they combined these discrete emotions into composite indices of low arousal positive affect, low arousal negative affect, high arousal positive affect, and high arousal negative affect, respectively, in their statistical analyses.

Since discrete emotions and dimensional affective states are theoretically distinct, people might accordingly be differentially accurate at predicting their intensity. Forecasting errors could be larger for dimensional states like valence and arousal since, according to discrete emotion researchers, these states are more abstract and nebulous, as compared to discrete emotions like fear that have universal, well-defined characteristics (Ekman, 1992). Alternatively, forecasting errors could be smaller for valence and arousal since, according to dimensional researchers, these states represent core affective processes that underlie the experience of any emotion (Barrett, 1998). Furthermore, people may find it easier to make predictions about the intensity of these core affective processes, as opposed to identifying, labeling, and predicting the intensity of individual discrete emotions.

In the same vein, it is likely that the processes underlying affective forecasting about discrete emotions are different from those that underlie affective forecasting about dimensional states. According to past research, the process of affective forecasting comprises three steps. First, people create mental simulations or “previews” of future events. Second, their previews induce hedonic reactions, or “premotions” in the present. Third, people then rely on the contexts that they are currently in and their simulations and premotions to create affective forecasts (Gilbert and Wilson, 2007, 2009). Discrete researchers could argue, for example, that people may be able to simulate situations involving discrete emotions more vividly than those involving positive/negative affect. This could also mean that they experience stronger premotions; if premotions accurately reflect the reality, this might lead to smaller affective forecasting errors for discrete versus dimensional states. Alternatively, dimensional researchers could argue that people may be able to easily simulate situations involving overall general feelings of positive/negative affect, rather than those involving specific discrete emotions. This could, in turn, induce stronger premotions, and lead to smaller affective forecasting errors for dimensional versus discrete states.

Although there are plausible hypotheses about why accuracy of dimensional versus discrete affective forecasts could be different, it is currently unknown whether accuracy actually is different. Prior to launching an in-depth investigation to understand whether measurement approaches could be a source of mixed findings, we need to assess whether there is in fact variation in measurement approaches in affective forecasting studies.

### 2.1 Current state of affective forecasting measurement approaches

In this paper, we conducted a mini review of peer-reviewed journal articles that have been published over the last few years.^2^ We only surveyed *recent* articles since our goal was to provide a snapshot of the current state of the affective forecasting literature, rather than to make broad claims about the entire affective forecasting literature. Understanding recent practices can illuminate fruitful research directions inspired by the researchers most likely to implement and expand on those directions. Our goal was to investigate to what extent recent studies of affective forecasting errors for emotion intensity are based on discrete or dimensional approaches. Furthermore, we examined *which* specific discrete emotions and dimensional affective states are most commonly examined in current research to reveal potential gaps in the literature. There were two key observations.

First, as seen in Tables 1, a majority of the studies published in the past few years adopted a hybrid approach in which they measured discrete emotions but ultimately analyzed these emotions as composite dimensional affective states, typically positive affect and negative affect. Only a few studies used a purely dimensional (e.g., measuring and analyzing valence), or purely discrete (e.g., measuring and analyzing happiness or fear) approach. A few studies used both discrete and hybrid approaches, and only one study used both pure discrete and pure dimensional approaches.

**Table 1:** Studies from the last few years (organized alphabetically) that measured affective forecasting errors for intensity of future emotions.

Study	Type of measure	Emotions/Affects	Findings for positive	Findings for negative
Aitken et al. (2021)	Discrete and dimensional	Excitement, Confidence, Pride, Nervousness, Frustration, Valence, Arousal	Underestimated Confidence, Pride; No significant forecasting error for Valence	Overestimated Nervousness, Frustration; Overestimated Arousal
Barber et al. (2023) (event: election win)	Hybrid	High Arousal Positive (HAP) Affect, High Arousal Negative (HAN) Affect, Low Arousal Positive (LAP) Affect, Low Arousal Negative (LAN) Affect	Overestimated HAP and LAP	Underestimated HAN and LAN
Barber et al. (2023) (event: election loss)	Hybrid	HAP, HAN, LAP, and LAN Affect	Underestimated HAP and LAP	Overestimated HAN and LAN
Buchanan et al. (2019) – Study 1	Discrete	Regret	-	Overestimated Regret
Carlson et al. (2022) – Study 1	Discrete	Happiness, Unhappiness	Overestimated Happiness (with as-expected grade outcome only)	Overestimated Unhappiness (with lower-than-expected grade outcome only)
Carlson et al. (2022) – Study 2	Discrete	Happiness, Unhappiness	No significant affective forecasting error	No significant affective forecasting error
Chanel et al. (2022)	Discrete	Fear, Anxiety, Excitement	Overestimated Excitement	Overestimated Fear, Anxiety
Colombo et al. (2020)	Hybrid	Positive and Negative Affect	Underestimated Positive Affect	Underestimated Negative Affect
Coundouris et al. (2022)	Discrete and Hybrid	Unhappy to Happy rating, Negative Affect	Underestimated Happiness	Overestimated Negative Affect
Dev et al. (2023)	Dimensional	Unhappy to Happy rating	-	Overestimated Negative Affect (unhappiness)
Study	Type of Measure	Emotions/Affects	Findings for Positive	Findings for Negative
Dillard and Meier (2023) – Study 1	Discrete and Hybrid	Regret, Guilt, Fear, Anger, Negative Emotion	-	Overestimated Regret, Guilt, Fear (no significant forecasting error for Anger); Overestimation of overall Negative Emotion
Dillard and Meier (2023) – Study 2	Discrete and Hybrid	Regret, Guilt, Fear, Anger, Negative Emotion	-	Overestimated Regret, Guilt, Fear, Anger; Overestimation of overall Negative Emotion
Dillard and Meier (2023) – Study 2	Discrete and Hybrid	Five discrete negative and five discrete positive emotions, Positive and Negative Affect		Overestimated Regret and Guilt only; Overestimated Negative Affect
Dorison et al. (2019) – Study 1	Hybrid	Positive Affect minus Negative Affect	-	Overestimated Negative Affect (net Positive Affect)
Frank et al. (2020)	Dimensional	Valence	Underestimated Positive Affect	Overestimated Negative Affect
Geiger et al. (2022)	Hybrid	Discomfort	-	Overestimated Discomfort
Holloway and Weiner (2021)	Dimensional	Positive and Negative Emotions	No significant affective forecasting error	Overestimated Negative Affect
Kaplan et al. (2020)	Hybrid	Positive and Negative Affect (confirmatory analyses)	Affective forecasting error for Positive Affect – but not directional	Affective forecasting error for Negative Affect – but not directional
Liu et al. (2022)	Hybrid	Positive and Negative Affect	Overestimated Positive Affect	Overestimated Negative Affect
Lu et al. (2022)	Discrete	Interest	Overestimated Interest	-
Mathersul and Ruscio (2020)	Hybrid	Positive / Negative Affect	Overestimated Positive Affect	Overestimated Negative Affect
Sekhsaria and Pronin (2021)	Discrete	Happiness	Underestimated Happiness	-
Study	Type of Measure	Emotions/Affects	Findings for Positive	Findings for Negative
Shovestul et al. (2022)	Hybrid	Positive and Negative Affect	Affective forecasting error for Positive Affect - but not directional	Affective forecasting error for Negative Affect - but not directional
Villinger et al. (2020)	Hybrid	Eating Happiness	Affective forecasting error for Eating Happiness – but not directional	-

Type of measure: we coded studies that used a dimensional approach to measure and analyze affective forecasting errors as “Dimensional.” This involved measuring dimensional states directly. We coded studies that measured discrete emotions but analyzed them as composite dimensional states as “Hybrid.” We coded studies that used a discrete approach to measure and analyze affective forecasting errors as “Discrete.” This involved measuring and analyzing each emotion as its own category. Emotions/Affects: we recorded the emotion/affect adjectives that researchers analyzed in their confirmatory analyses in each study. Findings for Positive and Negative: we recorded statistically significant findings from each study. Overestimation indicates that predicted emotions/affects < actual emotions/affects. Underestimation indicates that predicted emotions/affects > actual emotions/affects. If there was no statistically significant affective forecasting error, this is noted. If there was a statistically significant affective forecasting error, but it was not clear or specified what direction the error was in, this is noted as well.

Second, recent studies that adopted the discrete approach often examined discrete emotions that lie in the high arousal positive and negative affect quadrants (see Figure 1B). There is less recent work, however, on emotions that lie in the low arousal negative affect quadrant (barring one study that examined guilt and regret; Dillard and Meier, 2023), and almost no studies that examined emotions within the low arousal positive affect quadrant (barring one study that examined interest; Lu et al., 2022). Additionally, among the studies that we reviewed, there is relatively more granularity in the high arousal negative emotions quadrant than within any of the other quadrants, suggesting that researchers have neglected to examine emotions in the remaining three quadrants to the same extent.

Overall, there is variation in whether researchers adopt a discrete, dimensional, or hybrid approach in recent studies. Variation in approaches is, therefore, a factor worth considering as a systematic source of variation in the direction and/or magnitude of affective forecasting errors. Moreover, there are gaps in the recent literature regarding specific discrete emotions and dimensional affective states that suggest promising directions for further research. Filling these gaps can provide an understanding of affective forecasting errors for the wide range of emotions that people experience throughout their lives.

It should be noted that we purposefully surveyed only recent articles for this mini review. Thus, we cannot make broad claims about the affective forecasting literature going back more than 20 years. A systematic review of literature prior to 2019 could yield different conclusions. That said, we are unaware of a reason to expect that recent approaches to assessing forecasting errors are very different from past approaches. As such, the risk of our conclusions being biased seems low. Even if recent approaches are different from pre-2019 approaches, conclusions based on recent literature are arguably most relevant to identifying new directions for research by researchers actively working on this topic.

Also, we only included articles that reported affective forecasting errors for emotion intensity using an absolute accuracy approach rather than a relative accuracy approach (Mathieu and Gosling, 2012) and focused on non-clinical samples. We excluded studies that examined affective forecasts about factors other than intensity, those that assessed relative errors, and those that examined clinical populations. While it is certainly worth including studies that incorporated these features, such studies were outside the scope of the current review. Despite these caveats, our observations suggest a need for further systematic empirical investigations on the role of measurement approaches in affective forecasting errors as discussed below.

### 2.2 Future research directions

We offer three major directions for future research arising from our review of recent research. First, we found that there was variation in measurement approaches in affective forecasting studies (as seen in Table 1). However, one limitation of this review is that we cannot make inferences about whether affective forecasting accuracy differs *systematically* as a function of measurement approaches, especially given our focus on research published only in the past few years. Thus, empirical studies that directly compare forecasting errors assessed using discrete versus dimensional approaches are warranted. In addition, it appears there is reasonable variation to conduct a comprehensive meta-analysis of all the existing affective forecasting studies to determine whether the direction or magnitude of affective forecasting errors differs for dimensional versus discrete emotions/affective states. If such a meta-analysis reveals that errors for forecasts of affect are larger or smaller than those for discrete emotions, this could suggest that forecasting errors depend, in part, on the discrete versus dimensional state being forecast and, thus, are not exclusively marking trait-like differences in overall forecasting abilities. Additionally, it would encourage researchers to be more mindful of the measurements they collect, how they analyze results, and the inferences they draw.

Second, given the scarcity of studies examining granular dimensional affective states (apart from positive and negative affect) and discrete emotions that lie within the low arousal positive (such as contentment, serenity, and calmness) and negative (such as fatigue, tiredness, and boredom) quadrants, we recommend that researchers examine affective forecasting errors for these emotions and affective states. In fact, we recommend that researchers measure emotions or affective states that span the entire affective circumplex in their studies. In cases where researchers may only be interested in examining one discrete emotion for their confirmatory hypothesis, data on the remaining emotions can be explored and/or made openly available to other interested researchers. Collecting affective forecasting data about a variety of emotions and affective states will enable researchers to examine the robustness and reliability of the impact bias, uncover other trends across datasets, and better understand whether forecasting errors are specific to certain emotions/affective states.

Lastly, researchers should ask participants to make affective forecasts about a range of *events* that would likely induce emotions spanning the entire circumplex. In particular, forecasting studies often examine forecasts about focal events such as presidential elections (Dunn et al., 2007) and football games (Wilson et al., 2000) that likely induce high arousal emotions. Less common are studies that examine peoples’ affective forecasts about mundane events that likely induce lower arousal emotions such as completing tasks at work (e.g., Kaplan et al., 2020). However, given that people typically experience events that likely induce both low and high arousal emotions in their daily lives and that there is a lack of studies that have examined lower arousal emotions (as seen in Figure 1B), we recommend examining forecasting errors for a range of events that would induce emotions spanning the entire affective circumplex.

## 3 Conclusion

Despite the common claim that people make affective forecasting errors and, in particular, overestimate the intensity of their future emotions, evidence suggests that people sometimes underestimate or even accurately predict the intensity of their future emotions. In this paper, we suggest that discrete versus dimensional approaches to measuring affective forecasting errors could be a source of such variation in findings.

We reviewed studies published within the last few years and found that researchers vary in their use of dimensional versus discrete approaches to measuring and analyzing affective forecasting errors. However, our mini review was qualitative and, by design, too selective to make inferences about if or how discrete versus dimensional approaches affect the direction or magnitude of affective forecasting errors in the literature at large. Thus, we recommend conducting empirical studies that directly compare them and meta-analyses to examine whether affective forecasting errors differ systematically for dimensional versus discrete states.

Furthermore, recent studies that adopted a discrete approach often examined emotions in the high arousal positive (e.g., excitement) and negative (e.g., anger) affect quadrants of the affective circumplex. There is a lack of recent studies investigating more granular, dimensional affective states that span the entire affective circumplex (e.g., HAP, LAP, HAN, LAN) and discrete emotions in the low arousal positive (e.g., calmness) and negative (e.g., fatigue) affect quadrants. Thus, we recommend expanding the scope of affective forecasting investigations to examine the emotions/affective states that have not been examined previously and a variety of events that would likely evoke these emotions. Ultimately, the ideas we presented here will help researchers in the area design and conduct theoretically and methodologically sound affective forecasting studies, that will advance the field and provide a comprehensive understanding of affective forecasting errors for the intensity of future emotion.

## Author contributions

PP: Writing – original draft, Writing – review & editing. HU: Writing – review & editing.

## Footnotes

^1^Here we focus on affective forecasts about future emotion intensity since forecasts about intensity have been examined more extensively than forecasts about duration. Additionally, we focus on absolute, directional accuracy in this paper (the difference between predicted and actual affect), as opposed to relative accuracy (the correlation between predicted and actual affect), since forecasts using the former analysis technique have been examined more extensively than the latter.

^2^Inclusion criteria: (1) peer-reviewed articles in which participants rated both forecasted and actual affect within subjects, (2) keywords: “affective forecasting,” (3) publication years: 2019–2023. Exclusion criteria: (1) unpublished articles, (2) non-peer-reviewed theses/dissertations, (3) studies not focusing on forecasting emotion intensity, (4) studies that analyzed affective forecasting errors using the relative accuracy approach (Mathieu and Gosling, 2012; e.g., by conducting correlations/regressions between predicted and actual affect), and (5) studies of affective forecasting errors made by clinical populations.
